# Interdependent Transcription of a Natural Sense/Antisense Transcripts Pair (*SLC34A1/PFN3*)

**DOI:** 10.3390/ncrna8010019

**Published:** 2022-02-11

**Authors:** Hany S. Zinad, Chanachai Sae-Lee, Maria Ascensión Ariza-Mateos, Grace Adamson, Mushtaq Mufleh Khazeem, Amber Knox, Git Chung, Jelena Mann, Andreas Werner

**Affiliations:** 1 Biosciences Institute, Faculty of Medical Sciences, Newcastle University, Newcastle upon Tyne NE1 4HH, UK; dr.hany@nhm.uobaghdad.edu.iq (H.S.Z.); grace.adamson@live.com (G.A.); git.Chung@newcastle.ac.uk (G.C.); 2 Iraqi Natural History Museum and Research Centre, University of Baghdad, Baghdad 10071, Iraq; 3 Research Division, Faculty of Medicine, Siriraj Hospital, Mahidol University, Bangkok 73170, Thailand; chanachai.sae@mahidol.ac.th; 4 Department of Biochemistry and Molecular Pharmacology, Grossman School of Medicine, New York University, New York, NY 10016, USA; maria.arizamateos@nyulangone.org; 5 National Center of Hematology, Mustansiriyah University, Baghdad 10052, Iraq; mushtaqmufleh@gmail.com; 6 Newcastle Fibrosis Research Group, Biosciences Institute, Faculty of Medical Sciences, Newcastle University, Newcastle upon Tyne NE1 7RU, UK; amber4927@gmail.com (A.K.); jelena.mann@ncl.ac.uk (J.M.)

**Keywords:** natural antisense transcripts, concordant expression, transcriptional interference, CRISPR-Cas9, DNA methylation, histone modifications

## Abstract

Natural antisense transcripts (NATs) constitute a significant group of regulatory, long noncoding RNAs. They are prominently expressed in testis but are also detectable in other organs. NATs are transcribed at low levels and co-expressed with related protein coding sense transcripts. Nowadays NATs are generally considered as regulatory, long noncoding RNAs without closer focus on the inevitable interference between sense and antisense expression. This work describes a cellular system where sense and antisense transcription of a specific locus (*SLC34A1/PFN3*) is induced using epigenetic modifiers and CRISPR-Cas9. The renal cell lines HEK293 and HKC-8 do not express *SLC34A1/PFN3* under normal culture conditions. Five-day exposure to dexamethasone significantly stimulates sense transcript (*SLC34A1*) levels and antisense (*PFN3*) minimally; the effect is only seen in HEK293 cells. Enhanced expression is paralleled by reduced sense promoter methylation and an increase in activating histone marks. Expression is further modulated by cassettes that stimulate the expression of sense or antisense transcript but disrupt protein coding potential. Constitutive expression of a 5′-truncated *SLC34A1* transcript increases sense expression independent of dexamethasone induction but also stimulates antisense expression. Concordant expression is confirmed with the antisense knock-in that also enhances sense expression. The antisense effect acts on transcription in cis since transient transfection with sense or antisense constructs fails to stimulate the expression of the opposite transcript. These results suggest that bi-directional transcription of the *SLC34A1/PFN3* locus has a stimulatory influence on the expression of the opposite transcript involving epigenetic changes of the promoters. In perspective of extensive, previous research into bi-directionally transcribed *SLC34A* loci, the findings underpin a hypothesis where NATs display different biological roles in soma and germ cells. Accordingly, we propose that in somatic cells, NATs act like lncRNAs–with the benefit of close proximity to a potential target gene. In germ cells, however, recent evidence suggests different biological roles for NATs that require RNA complementarity and double-stranded RNA formation.

## 1. Introduction

Natural antisense transcripts (NATs) are long noncoding RNAs that are partly complementary to their protein coding sense counterparts. They are fully processed, i.e., spliced, polyadenylated and capped. NATs are predominantly expressed in testis, but also in somatic tissues, mostly together with the sense transcript and at low levels [1,2,3].

Bi-directionally transcribed genomic loci that encode complementary transcripts were discovered in the 1980s, though the scale of antisense transcription in all kingdoms, particularly in animals, only became apparent with the breakthrough in parallel sequencing strategies. Early pipelines to mine for NATs used bioinformatic tools to identify complementary sequences within comprehensive datasets or repositories [4]. To minimize the detection of false positive calls from reverse transcription artefacts, the input data were carefully parsed using hallmarks of RNA processing such as capping, splicing and polyadenylation. As a result, these early collections of NATs included predominantly fully processed mRNA-like transcripts. NATs are depleted from the X chromosome in humans and mice, suggesting a selection against complementarity exons [5,6,7]. Interestingly, the bias is not evident for transcripts from bi-directionally transcribed loci that hybridize as primary transcripts but not as processed RNAs, i.e., only display intron–exon complementarity. This observation suggests that at some point during the life cycle of NATs, formation of double-stranded RNA (dsRNA) occurs.

The number of bi-directionally transcribed loci detected in mammalian genomes crucially depends on specific experimental conditions and ranges between 40% and 70% [8]. Phylogenetic conservation for NATs is not clearly apparent and applies to indicators of bi-directional transcription per se, rather than primary sequence similarity [9,10]. Nevertheless, the prevalence of NATs and their intricate relation with protein coding genes have sparked countless efforts to decipher the regulatory potential of NATs. The relative stability of NATs allows for both transcriptional and post-transcriptional modes of action, and in both cases, stimulatory as well as inhibitory effects have been reported [8]. The topic has been reviewed extensively and will only briefly be touched here [2,11,12,13]. Activity on any of the DNA strands of a bi-directionally transcribed locus will lead to transcriptional interference, meaning that transcription on one strand influences the processivity on the opposite strand. Competition for transcription factors or transcription-dependent alteration of epigenetic marks generally leads to divergent expression levels of sense and antisense transcripts [13]. Conversely, if transcription on one strand facilitates access to enhancers/promoters on the other strand, a convergent sense/antisense relation may be observed. A scenario where two polymerase complexes are on collision course and ‘crash’ into each other is conceivable in yeast and bacterial model systems but unlikely to be of relevance in mammals [13].

NATs are fully processed mRNAs that can act as lncRNA either in the nucleus or the cytoplasm. NATs that serve as a platform for chromatin-modifying enzymes are widely documented [14]. Moreover, NATs often overlap with the 3′-UTR of the sense transcript and affect miRNA-mediated regulation of the sense transcript by masking binding sites or acting as a miRNA sponge [15]. Processed sense and antisense transcripts can also hybridize and form dsRNA [16], though the RNA hybrids come with a note of caution: Long stretches of dsRNA are reminiscent of viral structures that trigger an innate immune response and potential apoptosis [17]. Mammalian cells display a number of proteins that recognize dsRNA with different modalities and downstream effects, including ADAR (Adenosine Deaminase Acting on RNA), MDA-5 (Melanoma Differentiation Associated protein 5), Dicer and PKR (Protein Kinase R) [18]. In particular, ADAR, MDA-5 and PKR have recently been shown to exert a protective function against endogenous dsRNA formed in the mitochondria and the nucleus from the transcription of repetitive elements [19,20]. NATs-related dsRNAs have not been investigated in this context, but are unlikely to reach a level high enough in somatic cells to trigger an antiviral response. Similarly, endogenous siRNAs (Dicer products) have been sequenced and mapped without significant enrichment in sense/antisense complementary regions [21]. The situation is different in male germ cells, where NATs as well as endo-siRNAs were recently shown to be linked to loci that form dsRNA [16]. These findings are in line with reports of bi-directionally transcribed loci that give rise to endo-siRNAs in testis, one of which is the *SLC34A1/PFN3* gene (Solute Carrier 34A1/Profilin 3) [22,23,24].

The *SLC34A1* gene encodes an epithelial sodium-dependent phosphate transport system crucially involved in balancing whole body phosphate levels. It is expressed almost exclusively in renal proximal tubules, and the major hormones known to maintain phosphate homeostasis (parathyroid hormone and fibroblast growth factor 23) regulate its presence in the brush border membrane. These hormones acutely affect protein localization and stability rather than significantly impacting on transcription, which is stimulated in response to low phosphate levels [25]. The *PFN3* gene is localized immediately downstream of *SLC34A1* in opposite orientation, and alternative splicing generates a noncoding, overlapping antisense transcript (Section 2.1) [7].

The various facets of NATs, some emerging only recently, have shaped the perception of antisense RNA biology over time. In the early days, NATs were considered a family of transcripts on their own, with spatially restricted modes of action in conjunction with related sense transcripts and genes. Moreover, features were established that applied to NATs in general, such as expression levels, expression patterns, X-chromosome bias, phylogenetic conservation and intron size [3,26]. With the ever-increasing sequencing depth and emergence of thousands of long non-coding RNAs (lncRNAs) that share certain features of NATs, the general conception of antisense RNAs has shifted and they are now considered as lncRNAs, without taking the distinct features of NATs into account.

We have studied NATs in a genomic context and *SLC34A*-related antisense regulation in various model systems. For example, we have investigated antisense transcripts at a genome level in cell lines (HEK293 cells), somatic tissues and germ cells [16,21,22]. Moreover, the generation of endo-siRNAs from *Slc34a* sense/antisense transcripts was characterized in *Xenopus* oocytes, though we could not detect *SLC34A*-derived short RNAs in HEK cells [21,22]. Here, we investigate the interplay between the *SLC34A* sense/antisense pair, including the epigenetic state of the locus, and extrapolate the findings to suggest common principles of gene regulation by NATs.

## 2. Results

### 2.1. The SLC34A1/PFN3 Locus

**Figure 1:**
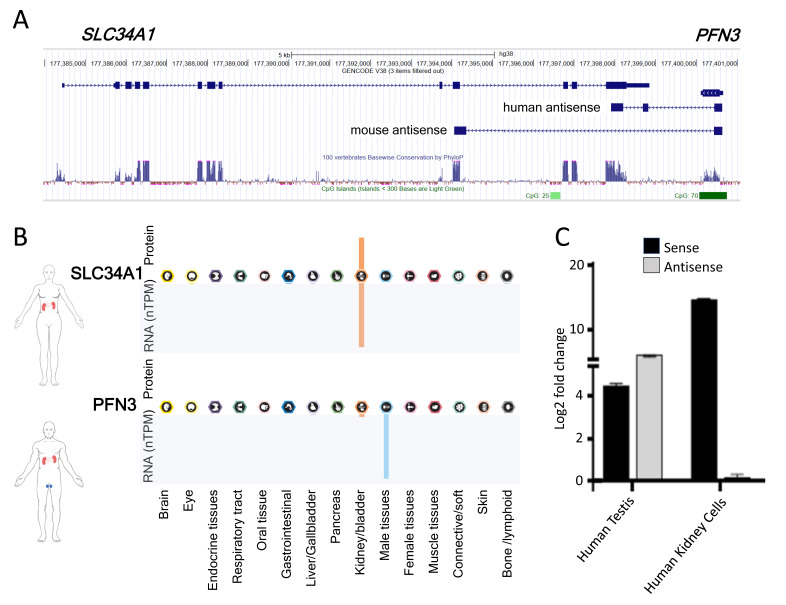
Architecture and expression of the *SLC34A1/PFN3* (sense/antisense) locus. (**A**) Modified screenshot of the human *SLC34A1/PFN3* locus in the UCSC genome browser. The *SLC34A1* sense gene and the *PFN3* gene in antisense are at the top, published human [7] and murine [23] antisense transcripts are below. Conservation in 100 vertebrates (light blue) and CpG islands (green). (**B**) Expression of *SLC34A1* and *PFN3* mRNA and protein in various organs adapted from the human protein atlas (https://www.proteinatlas.org/, accessed on 10 December 2021). (**C**) Expression of sense (*SLC34A1*, black bars) and antisense transcripts (*PFN3*, grey bars) was assessed by RT-qPCR in human testis RNA and RNA extracted from primary kidney cells. The log2 fold change refers to uninduced HKC-8 cells.

The *SLC34A1* gene is well conserved, and its exon/intron structure is identical in all mammals (Figure 1A). Moreover, three homologues (*SLC34A1*, *SLC34A2* and *SLC34A3*) exist with comparable genomic and protein structures, and distinct tissue distribution that reflects their physiological function. *SLC34A1* mRNA is detectable in human testis but at very low levels compared to the kidney (Figure 1B,C).

The *PFN* gene family contains four members, with profilin 1 and 2 displaying close similarity (>60% homology) but PFN3 and PFN4 only sharing <40% and <20% amino acids with other Profilin isoforms [27]. The predicted 3D structures of all isoforms, however, are clearly related (https://alphafold.ebi.ac.uk/, accessed on 10 December 2021). What separates *PFN3* from the other *PFN* genes are the poor phylogenetic conservation, the lack of introns and mRNA expression restricted to testes (Figure 1A,B). In addition, large scale proteomics approaches did not detect PFN3 protein in any of the assessed tissues, including testis (Figure 1B). In previous work, we cloned *PFN3*-related 3′RACE products from human and mice testes and only found alternatively spliced transcripts that resulted in a truncated *PFN3* open reading frame and ran into the *SLC34A1* gene, generating a natural antisense transcript (Figure 1A) [7,23]. Hence, the non-protein coding antisense transcript constitutes a significant proportion of the *PFN3* transcriptional output.

There is currently no epithelial human cell line with an active *SLC34A1/PFN3* locus; the only cell line that expresses *Slc34a1* is derived from opossum kidney (OK cells) [28]. Moreover, primary cells from human kidneys lose the expression of the transporter within about two weeks in culture [29].

### 2.2. Drug-Induced Expression of SLC34A1/PFN3 Sense-Antisense Transcripts

**Figure 2:**
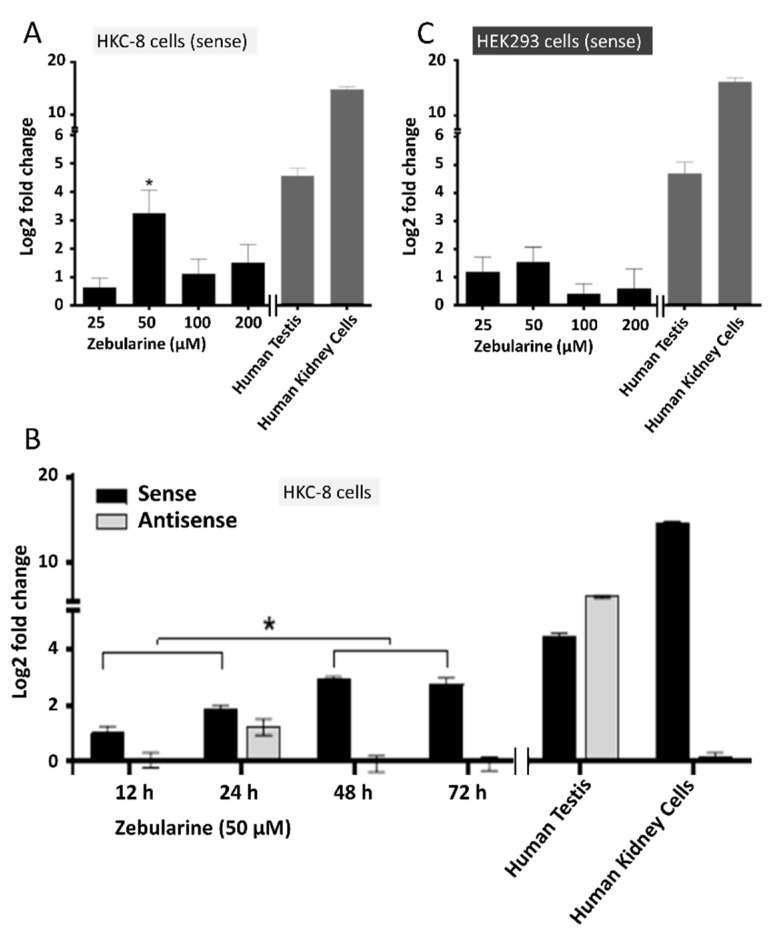
Expression of the *SLC34A1/PFN3* (sense/antisense) locus in in response to zebularine. The expression of sense (*SLC34A1*) and antisense transcripts (*PFN3*) was assessed by RT-qPCR; human testis RNA and RNA extracted from primary kidney cells were used as controls (right panels of (**A**–**C**)). (**A**) Response of the sense transcript to zebularine in HKC-8 cells and (**B**) HEK293 cells. (**C**) The most effective dose was used (50 µM) to establish the time course of expression for both the sense (black) and the antisense (grey, only detectable at 24 h) transcripts. Statistical significance was tested by one-way ANOVA followed by Tukey’s test for multiple comparisons, * *p* < 0.05.

Natural sense/antisense transcripts are often co-expressed in the same RNA preparations [30]. To assess potential co-expression *SLC34A1/PFN3* transcripts, we used the established human kidney cell lines HEK293 and HKC-8, and drugs that modify the epigenetic imprint to identify conditions that lead to detectable transcripts from the *SLC34A1/PFN3* locus. Cells were exposed to zebularine (DNA methyltransferase inhibitor), trichostatin A (histone deacetylase inhibitor) and dexamethasone (broad effect on both DNA methylation and histone modifications) followed by expression analysis of *SLC34A1/PFN3* sense and antisense transcripts by RT-qPCR. The primers used to amplify fragments of *PFN3* only detected the splice form that overlaps the *SLC34A1* gene. In HKC-8 cells, the *SLC34A1* sense transcript was significantly enhanced in response to zebularine as compared to non-treated cells; dose-response experiments established 50 µM zebularine as the most effective dose (Figure 2A).

The increase in HKC-8 cells was time-dependent, and it plateaued after 48 h incubation (Figure 2B). A comparable response was measured in HEK293 cells, but the increase in mRNA was not significant (Figure 2C). The *PFN3* antisense transcript was only minimally expressed in HK-8 cells, and detectable after 24 h incubation with the drug (Figure 2B). Trichostatin A, on the other hand, did not provoke any detectable output from the *SLC34A1/PFN3* locus, despite global acetylation levels rising significantly after drug treatment (Figure S2). Moreover, trichostatin A and zebularine showed no signs of synergistic stimulation (not shown).

**Figure 3:**
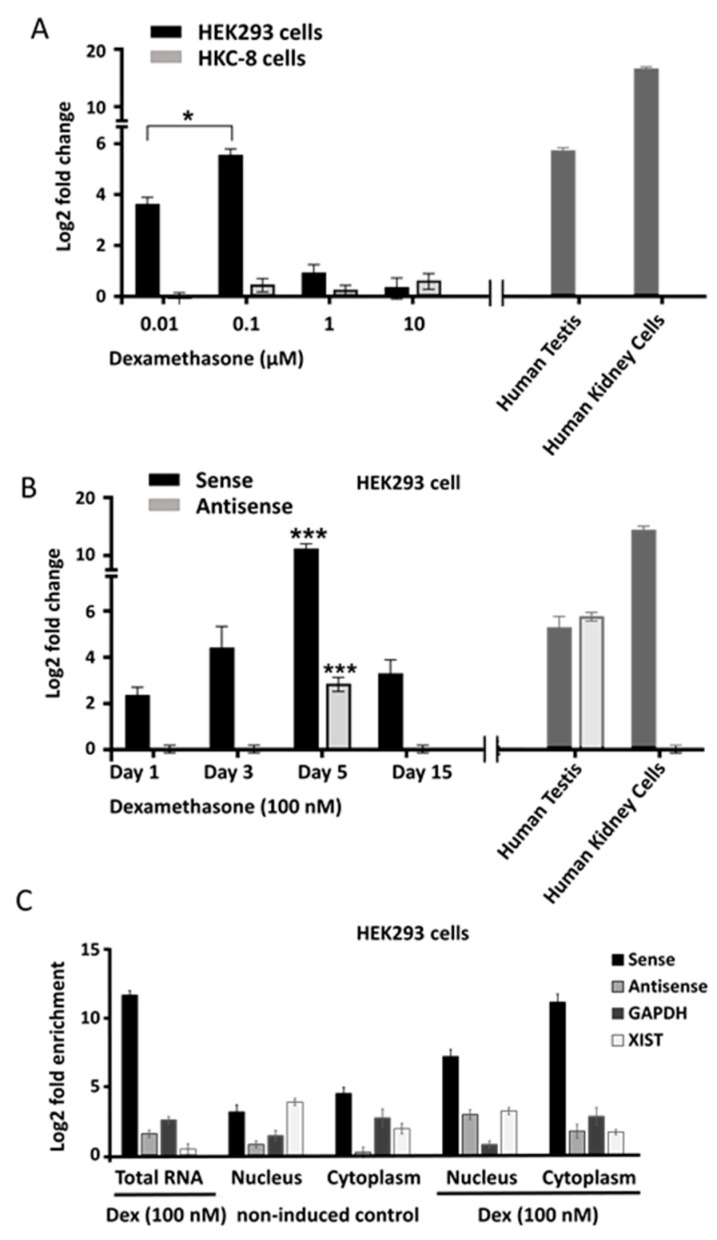
Expression of sense and antisense transcripts in response to dexamethasone in HEK293 and HKC-8 cells. (**A**) Response of the sense transcript to increasing levels of dexamethasone in HEK293 cells (black) and HKC-8 cells (grey) measured by RT-qPCR. Human testis RNA and human kidney cell RNA were used as positive controls (right panel). (**B**) Time dependence of the dexamethasone response of sense (black) and antisense (grey) transcripts in HEK293 cells. Control RNAs from testis and kidney on the right. (**C**) Nuclear and cytoplasmic distribution of sense and antisense transcripts in HEK293 cells. Nuclear and cytoplasmic fractions were enriched followed by RNA extraction and RT-qPCR from control and dexamethasone induced cells (underscored). *XIST* was measured to estimate nuclear enrichment, *GAPDH* for cytoplasmic enrichment. One-way ANOVA followed by Tukey’s test for multiple comparisons, * *p* < 0.05; *** *p* < 0.001.

Dexamethasone (100 nM) provoked the most prominent transcriptional stimulation of the *SLC34A1/PFN3* locus in HEK293 cells; a comparably small response was observed in HKC-8 cells under identical conditions (Figure 3A).

The drug increased the expression of the sense transcript in a time-dependent manner up to about 15-fold after five days; in parallel, antisense transcript levels were also enhanced by approximately 3-fold (Figure 3B). Separation of nuclear and cytoplasmic cell fractions revealed enrichment of the sense transcript in the cytoplasm whereas the small amount of antisense transcript was confined to the nucleus (Figure 2C). The sense transcript followed the enrichment pattern of the cytoplasmic control *GAPDH* whereas the antisense transcript paralleled *XIST* nuclear enrichment.

### 2.3. Epigenetic Changes in Response to Zebularine and Dexamethasone

**Figure 4:**
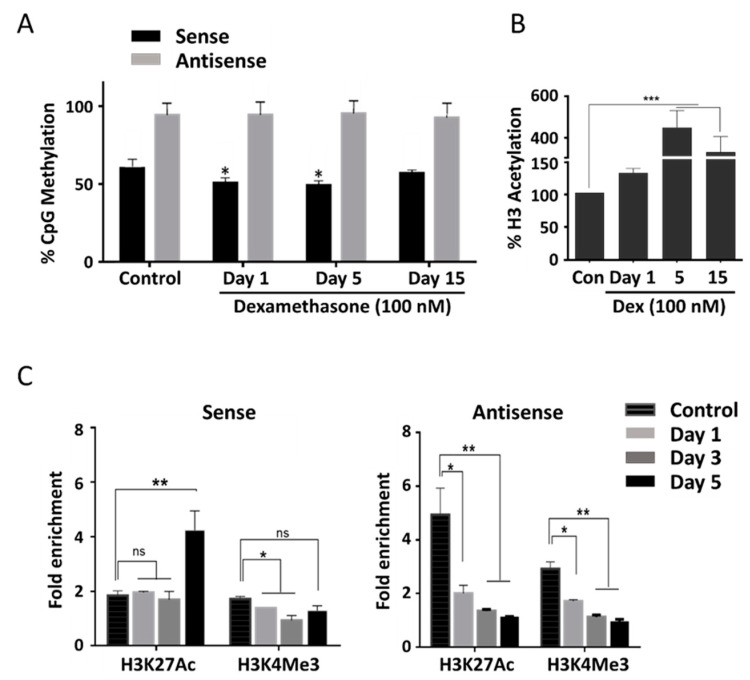
DNA methylation and histone H3 modifications of *SLC34A1/PFN3* sense and antisense promoters in HEK293 cells. (**A**) DNA methylation levels of 7 CpGs in the sense promoter (black) and 6 CpGs in the antisense promoter (grey) after exposure to dexamethasone for 1, 5 and 15 days. Fully methylated (100%) and un-methylated fragments (0%) served as controls, methylation levels in cells exposed to dexamethasone for 1, 5 and 15 days were compared to cells without the drug. (**B**) Global histone H3 acetylation in response to the exposure of dexamethasone for 1, 5 and 15 days. (**C**) Chromatin immunoprecipitation (ChIP) using antibodies against H3K27Ac and H3K4Me3 followed by qPCR of sense (left panel) and antisense (right panel) promoters. Cells were exposed to dexamethasone for 1, 3 and 5 days or left without the drug as a control. Significance was established using one-way ANOVA and Tukey’s test for multiple comparisons, * *p* < 0.05; ** *p* < 0.01; *** *p* < 0.001; ns = no significance.

The promoter regions of both *SLC34A1* sense and *PFN3* antisense genes contain a short CpG island with seven and six CpGs, respectively. We tested whether zebularine and dexamethasone had an effect on the methylation status of both sense and antisense promoters (Figure 4A and Figure S1). Quantitative pyrosequencing revealed that zebularine induced small but significant sense promoter hypomethylation that parallels the expression changes in HKC-8 cell lines (Figure S1). A comparable reduction in sense promoter methylation was seen with HEK293 cells; in contrast, the antisense promoter stayed fully methylated (Figure 4A).

To investigate further the epigenetic changes induced by dexamethasone, chromatin immunoprecipitation (ChIP) was performed with HEK293 cells. First, global H3 histone acetylation was shown to significantly increase by about four-fold in response to dexamethasone treatment. This effect peaked at 5 days of treatment and became reduced after 15 days but was still significantly above control (Figure 4B). To characterize histone modifications in the *SLC34A1/PFN* promoter regions, chromatin immunoprecipitation (ChIP) was performed using antibodies against H3K27Ac (acetylated lysine 27 of histone H3) and H3K4Me3 (tri-methylated lysine 4 of histone H3) followed by qPCR of the *SLC34A1/PFN* promoter regions. Both histone marks reflect active promoters.

H3K27Ac in the sense promoter region clearly increased at day 5 in response to dexamethasone whereas H3K4Me3 remained largely unaffected. In contrast, both histone marks in the antisense promoter decreased over time and showed their lowest level after 5 days of dexamethasone (Figure 4C).

To summarize, transcription at the *SLC34A1/PFN3* locus can be activated using zebularine and dexamethasone, but the effect crucially depends on the cell lines used. The activation is generally stronger for the *SLC34A1* sense transcript and only just detectable for the antisense RNA. Epigenetic remodeling in response to the drugs supports the increased transcriptional activity for the sense transcript, in contrast, the antisense promoter did not display active chromatin marks. These findings support a hypothesis where dexamethasone induces transcription of the sense transcript which, in turn, stimulates antisense transcription.

### 2.4. Genetic Interference with SLC34A1/PFN3 Sense–Antisense Expression

To scrutinize this hypothesis, we aimed to modulate *SLC34A1/PFN3* sense and antisense transcription individually by means of CRISPR-Cas9-mediated insertion of transcriptional termination cassettes. HEK293 cells were used for the knock-in of a transcription termination cassette.

**Figure 5:**
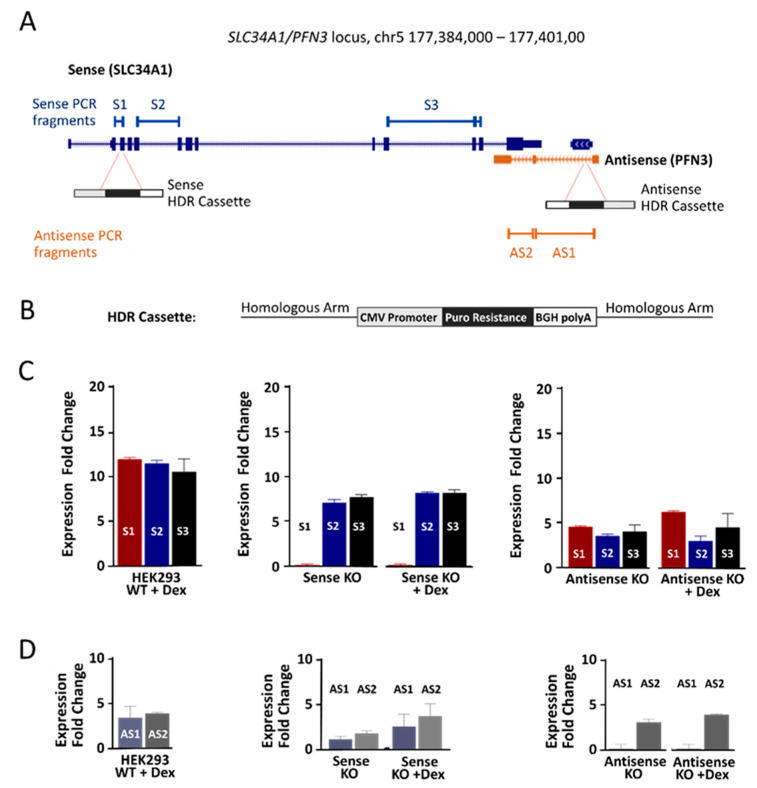
Constitutive low-level activation of sense and antisense expression in HEK293 cells. (**A**) Snapshot of the *SLC34A1/PFN3* locus with the insertion sites for the HDR (homology-directed repair) cassettes and primer sites S1–S3 as well as AS1 and AS2. (**B**) Schematic representation of the HDR cassette containing a CMV promoter, the puromycin resistance, the BGH polyadenylation signal and the two gene-specific flanking regions (homologous arm). The cassette was meant to shut down transcription, but insertion produced low levels of read-through transcripts driven by the CMV promoter. (**C**) Sense transcript expression in cells with the knock-in construct in sense orientation (middle) and antisense orientation (right). Wildtype HEK293 cells stimulated with dexamethasone (left) were used as positive controls; all expression levels were referred to unstimulated HEK293 wildtype cells. The bars represent specific primer pairs and are color coded, S1 red, S2 blue and S3 black. Of note, primer pair 1 is upstream of the cassette insertion site and does not amplify the read-through transcript. (**D**) Antisense transcript expression in CRISPR edited HEK293 cell clones, the left panel shows unedited HEK293 cells exposed to dexamethasone as a control. Monoallelic insertion of the cassette placed in sense (middle) and antisense (right) orientation. The primer pair AS1 (blueish) flanks the cassette and only generates a PCR product from clones without an insertion. Data are the mean from three independent biological and three technical replicates. Expression levels are normalized to *GAPDH*, and the fold change (2^−ΔΔCT^) was calculated in comparison to the non-treated HEK293 cells.

Guide RNAs were designed to direct Cas9 to the second (sense) and the first intron (antisense), respectively (Figure 5A). Cassettes were prepared containing the CMV promoter driving a selection marker that confers resistance to the antibiotic puromycin in addition to a strong polyadenylation signal (BGH) and specific sequences flanking the double strand breaks to direct homologous DNA repair (Figure 5B) [31]. Biallelic insertions were achieved with a single homology template, thanks to highly efficient Cas9. The presence of both mono- and bi-allelic insertions in individual clones was tested by PCR (Figure S3), and clones with bi-allelic insertion were used for further studies.

First, the consequences of knock-ins on *SLC34A1/PFN3* expression were tested using various primer pairs downstream of the insertion sites. Unexpectedly, significant transcript levels could be detected for sense (S2 and S3 in the middle panel of Figure 5C) and antisense (AS2 in the right panel of Figure 5D), indicating that the promoter driving the antibiotic resistance gene produced read-through transcripts into the opposite gene. Intriguingly, the read-through transcripts provided a tool to differentiate between transcriptional activation by dexamethasone and transcriptional interference, where transcription in one direction alters the RNA levels of the complementary transcript.

The level of *SLC34A1* read-through RNA was 7–10 times (Figure 5C middle, S2 and S3), the *PFN3* truncated transcript 3–5 times (Figure 5D, right panel) over the background of non-edited, non-stimulated HEK293 cells. The small difference in sense and antisense transcripts may reflect different inherent promoter activity of sense/antisense or the stability of the two RNAs. Importantly, the level of read-through transcripts mirrors the increased expression of sense and antisense RNAs in response to dexamethasone. The consequences of the read-though transcripts on RNA levels from the opposite gene were tested by RT-qPCR: Transcription from either direction leads to a significant threefold increase in the respective complementary mRNAs (antisense-induced sense expression, Figure 5C right panel; sense-induced antisense expression, Figure 5D, middle panel). Comparable experiments were performed with knock-in cells after exposure to dexamethasone. However, the effects of the drug were almost completely blunted by the presence of the cassette, regardless of the direction of its transcription.

To test whether the positive interference between sense and antisense transcript was acting in cis, we transiently transfected HEK293 cells with PCR fragments encoding each of the two transcripts (50 and 250 ng/mL). 

**Figure 6:**
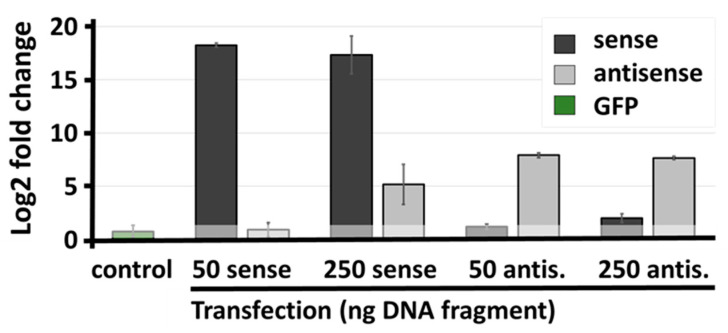
Transient expression of sense/antisense cassettes in HEK293 cells. Cells were transfected with two doses (50 and 250 ng/mL) of cDNA encoding, either the sense transcript (dark grey) or the antisense transcript (light grey). Expression levels of sense and antisense transcripts were assayed by RT-qPCR to detect potential post-transcriptional stabilizing effects. All values are related to untransfected HEK293 cells. To estimate PCR background, GFP was amplified in all samples (control and shaded area reflecting non-significant changes).

At 50 ng/mL, neither sense nor antisense transgene stimulated the expression of the opposite transcript (Figure 6). The higher amount of DNA appeared to over-saturate the cells since the expression of transgene was not further increased compared to the lower dose. The complementary transcript appeared to become stabilized, though this effect may be unspecific.

### 2.5. Protein Expression

The mRNA for *SLC34A1/PFN3* sense and antisense was found in both nucleus and cytoplasm with moderate enrichment for the transporter-encoding transcript in the cytoplasm (Figure 3C). Hence, we investigated whether the increased levels of *SLC34A1* mRNA gave rise to functional Na/Pi transport protein.

**Figure 7:**
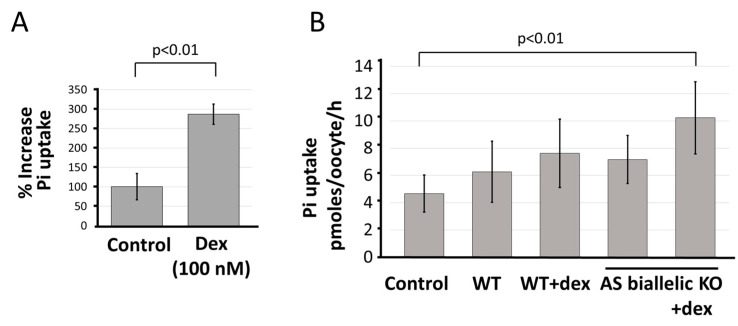
Functional expression of *SLC34A1* in *Xenopus* oocytes and HEK293 cells. (**A**) Wild type HEK293 cells were grown with and without dexamethasone for 5 days, followed by uptake measurement using radioactive Pi. Values were scaled to an average control value of 100%, *t*-test revealed significance, *p* < 0.01. (**B**) RNA was extracted from HEK293 cells, untreated (WT) and after 5 days exposure to dexamethasone (WT + dex). The same procedure was carried out with HEK293 cells that had biallelic insertion of the antisense cassette (AS biallelic KO + dex). Total RNA was injected into *Xenopus* oocytes, and the uptake of radioactive Pi was measured after 3 days. One-way ANOVA followed by Tukey’s test for multiple comparisons, *p* < 0.01.

Two different experimental approaches were pursued, including epithelial cell uptake and functional expression, in *Xenopus laevis* oocytes. The experiments revealed low expression of the protein/function that was close to detection level. HEK293 cell monolayers were treated with dexamethasone for 5 days, and tested for the uptake of radioactive phosphate. There was phosphate uptake through unspecified carriers producing significant background, nevertheless, stimulation of *SLC34A1* expression through dexamethasone approximately doubled the transport of phosphate (Figure 7A). To obtain further confirmation that the dexamethasone stimulated output from the *SLC34A1/PFN3* locus was biologically active, we purified total RNA from wild type and knock-in cells (WT, WT + dex, antisense knock-in, antisense knock-in + dex). The mRNA was injected into *Xenopus* oocytes, followed by uptake measurements after 3–4 days. Again, the effects were small but a stimulatory effect of both knock-in and dexamethasone treatment was observed (Figure 7B).

To summarize, we have shown that the *SLC34A1/PFN3* sense-antisense transcripts interfere at the level of transcription in a cell-specific manner. We propose a model whereby dexamethasone stimulates the expression of the transporter-encoding sense gene. Altered chromatin marks parallel the transcription on the sense strand which, in turn, promotes low-level transcription of the *PFN3*-antisense gene. The concordant interaction is active in cis but not in trans. The increased transcript levels appear to have a minor impact on protein expression.

## 3. Discussion

We report a cell culture system that allows manipulating the expression of the *SLC34A1/PFN3* sense–antisense transcript pair using dexamethasone and CRISPR gene editing. In essence, we found that sense and antisense transcripts are positively co-regulated, and mRNA levels are paralleled by epigenetic marks, i.e., DNA methylation and histone modifications in sense but not antisense direction. The observed effects are cell line specific, as generally observed with other regulatory lncRNAs.

The most striking feature of natural antisense transcripts is their obvious regulatory potential related to complementary sense transcripts. Hence, a sense-antisense arrangement of gene pairs was hypothesized to provide an evolutionary advantage, possibly through one of a few specific, conserved molecular mechanism(s) [12,32]. In fact, several early observations suggested that such antisense-mediated regulatory mechanisms indeed existed. For example, sense and antisense transcript pairs were found in the same cDNA libraries [6]. Convergent genes show hallmarks of evolutionary conservation [9] and potentially common structural features [26], and natural antisense transcripts are depleted in the mammalian X chromosome [5,6]. However, testable predictions deduced from hypothesized regulatory mechanisms, for example specific functional or structural properties of bi-directionally transcribed genes or endo-siRNA formation, gathered only limited experimental support [33,34]. Output and outcome of sense–antisense transcription and transcript interactions depend largely on the model system where the co-expression is studied. Both stimulatory and inhibitory effects of antisense transcripts on sense transcript expression are now widely documented, as is independent regulation of the two complementary genes [8,35].

We report a cell culture model with inducible sense and antisense gene expression. With both dexamethasone stimulation and CRISPR-mediated transcriptional activation, convergent expression of sense and antisense RNAs was observed. The observed effects only became detectable after extended incubation time (5 days), though other systems have been reported where epigenetic changes and transcription of the affected locus were detected in a much shorter time frame of minutes [36]. The effect of dexamethasone was shown to be dependent on transcription rather than RNA-mediated, since trans-activation from transfected DNA constructs was not observed. Moreover, comparable effects elicited by different stimulatory interventions (dexamethasone and CRISPR) suggest that transcription itself rather than an interaction of the promoters provides the stimulatory drive. The activation was reflected by reduced promoter methylation and activating histone marks (H3K27Ac) at the sense promoter, less so at the antisense promoter. Accordingly, the stimulatory effect of dexamethasone and sense transcription on antisense expression was weak. Coordinated expression of clustered genes is a well-documented phenomenon, originally described as ‘transcriptional ripples’ [37,38]. The transcriptional co-regulation of locally connected genes often does not feed through to the protein level, i.e., transcriptome changes are not reflected at the proteome level [39]. Our observations that the *SLC34A1*-encoded phosphate transporter was difficult to detect in phosphate uptake experiments indicate that also in our HEK293 cell model, the *SLC34A1* mRNA is not efficiently translated. The levels of *PFN3* mRNA are even considerably low in HEK293 cells and localized in the cell nucleus; nevertheless, we cannot rule out that the *PFN3* gene is protein coding. In fact, immunological detection of PFN3 protein has been reported in mouse testes in the final stages of sperm development [40]. Moreover, disruption of *Pfn3* causes abnormal acrosome formation and leads to subfertile male mice [41].

Antisense transcripts complementary to *SLC34A* loci have been studied in various model systems, including zebrafish embryos, *Xenopus* oocytes, mice and human cell lines [7,22,23,42]. Initial expression analyses focused on testes and kidneys where the antisense and sense transcripts, respectively, are predominantly detected [43]. However, evidence that an antisense-mediated mechanism contributes to the physiological regulation of the sense-encoded phosphate transporter and phosphate homeostasis proved inconclusive. Accordingly, *SLC34A* sense and antisense transcripts show different expression patterns; the sense transcripts are present in epithelial tissues of the kidney (*SLC34A1*) and intestine (*SLC34A2*, mammals and fish), and the antisense RNA is most prominently expressed in testis [44,45]. Co-expression has been detected during zebrafish development and also in mouse testis and kidney, though the biological rationale for the expression may not be the related to Pi homeostasis (see below) [22]. During a window of co-expression in zebrafish, endo-siRNAs were detected, pointing to an antisense mediated regulatory mechanism that involves dsRNA formation and processing by Dicer [46]. However, this mechanism appears to not apply to the cell culture model described here, since the gene pairs producing endo-siRNAs are usually antagonistically regulated and we were unable to detect *SLC34A1/PNF3*-derived small RNAs on Northern blots (not shown).

In the early days of the genomic era, NATs were considered a separate family of non-protein coding transcripts, distinct from other long noncoding RNAs with a specific biological rationale and potentially common mechanistic themes. The mechanisms discussed, i.e., the various modes of transcriptional interference and post transcriptional sense–antisense interactions, have been briefly outlined in the introduction and are extensively reviewed [3,11,13]. An increasing number of recent studies have focused on mechanistic aspects of sense and antisense expression, especially in the context of cancer. Two types of strategies clearly prevailed: The function as a microRNA sponge and an interaction of the antisense transcript with chromatin-modifying enzymes. The importance of the former mechanism has only emerged in the past few years, predominantly in the context of cancer. A common experimental strategy identifies a differentially expressed antisense transcript in cancer vs. untransformed cells followed by miRNA target gene prediction and experimental validation [14], ideally demonstrating the direct interaction between miRNA and target antisense transcript [47]. Alternatively, immunoprecipitation of Ago2 followed by RT-qPCR was used to corroborate the interaction between the miRNA and its target antisense transcript [48].

The regulatory mechanism where antisense transcripts induce chromatin modifications prominently involves polycomb repressive complex 2 (PRC2). PRC2 induces H3K27 trimethylation [49], which could relate to the decrease in H3K27 acetylation that we observed at the antisense promoter in response to dexamethasone (Figure 4) [50]. Molecular aspects of PRC2-mediated gene silencing are well established in the context of X chromosome inactivation and the noncoding RNAs *Xist* and *Tsix*, the antisense transcripts *Airn* and Kcq1ot1 in parental imprinting, as well as the antisense lncRNAs *Hotair* and *Anril* [51,52,53]. The specific mechanism depends on the interaction of PRC2 with RNA molecules as well as interactions with additional proteins and phase separation. Sequencing of PRC2-bound RNA from mouse embryonic stem cells identified 4224 transcripts (43.2%) antisense to annotated UCSC transcripts [54]. The observation that other lncRNA bind to PRC2 equally well suggests that antisense transcripts are correctly considered as a subfamily of lncRNAs, possibly acting locally and preferentially targeting the sense gene.

On the other hand, few recent findings support common themes for antisense transcripts in early zebrafish embryos and during germ cell development [16,55]. In zebrafish embryos, natural antisense transcripts that are inversely regulated compared to their sense partner are associated with developmental genes, and are hypothesized to suppress spurious expressing during early zebrafish development [55]. Experiments with mouse testis found that the expression of natural antisense transcripts was confined to a specific developmental stage (pachytene spermatocytes). Moreover, there is evidence that sense and antisense transcripts form dsRNA and are further processed into endogenous siRNAs. It is hypothesized that antisense transcription makes part of a control mechanism which parses germ cells that suffered a deleterious outcome after recombination and transposon mobilization [16,56].

## 4. Materials and Methods

Cell culture: HEK293 cells were a kind gift from Nicholas Watkins (Newcastle) and originally purchased from Invitrogen/Thermo Fisher (Waltham, MA, USA). HKC-8 cells were obtained from Prof. Neil Sheerin (Newcastle) and maintained at the Cell Bank, Biosciences Institute, Newcastle University. HEK293 cells were cultured in DMEM-high glucose and HKC-8 cells in DMEM/HAM-F12 medium, supplemented with 10% fetal calf serum (FCS), 2 mM L-glutamine, 1% non-essential amino acids, 100 U/mL penicillin and 100 µg/mL streptomycin. Cell culture media and supplementary reagents were purchased from Sigma (St. Louis, MO, USA). Cells were seeded 24 h prior to treatment in six well plates at 0.8 × 10^6^ cell density. Zebularine, dexamethasone (Abcam, Cambridge, UK) and trichostatin A (Sigma) were used at various indicated concentrations.

Gene expression profiling: Total RNA was extracted using a Quick-RNA Miniprep Plus Kit (Zymo Research, Orange, CA, USA) including on-column DNA digestion according to the supplier’s instructions. RNA was quantified using Nano drop (Thermo Fisher, Waltham, MA, USA) and the integrity was confirmed by 2% agarose gel electrophoresis. Total RNA from kidney primary cells was obtained from Newcells (Newcastle, UK), who grow the cells from normal human kidneys according to Bajaj et al. [57]. Human testis RNA was purchased from Takara (Kusatsu, Japan). For the RT-qPCR experiments, the Luna Universal One-Step RT-qPCR kit (New England Biolabs, Ipswich, MA, USA) was used. Samples contained 100 ng of total RNA for *SLC34A1*-sense/antisense transcripts and 25 ng for *GAPDH*. Reactions were run on a LightCycler^®^ 480 system (Roche, Basel, Switzerland) with the following parameters: Reverse transcription at 55 °C for 10 min; initial denaturation at 95 °C for 1 min; denaturation at 95 °C for 10 s and extension at 60 °C for 30 s for 45 cycles. RNA from primary renal epithelial cells and human testis RNA were included as positive controls. All primers used for the RT-qPCR and other experiments were synthesized by Integrated DNA Technologies (Coralville, IA, USA), and are listed in Tables S1–S6. The expression is given as the fold change (R) of treated vs. non-treated cells calculated as ΔΔCt = ΔCt (non-treated) − ΔCt (treated).

*Pyrosequencing:* Genomic DNA was purified using the Wizard^®^ Genomic DNA Purification Kit (Promega, Madison, WI, USA). In total, 450–500 ng of gDNA was subjected to sodium bisulfite treatment to deaminate un-methylated cytosines to uracils using the EZ DNA Methylation Gold Kit (Zymo Research). The promoter regions were PCR amplified using forward and biotinylated reverse primers (Table S2). PCR products were examined on a 2% agarose gel and confirmed by Sanger sequencing (GATC Biotech, Ebersberg, Germany). PCR amplicons were bound to Streptavidin Sepharose High Performance beads (GE Healthcare, Chicago, IL, USA), washed with 70% ethanol, PyroMark Denaturation Solution and PyroMark Washing buffer (Qiagen, Hilden, Germany), followed by annealing of the sequencing primers. Pyrosequencing reactions were run on a PyroMark Q96 ID (Qiagen). A positive control (100% methylation) was generated with 10 U of CpGs methyltransferase (*M.Sss*I) (New England Biolabs) and unmethylated DNA (0% methylation) using a whole genome amplification kit (Qiagen).

Chromatin immunoprecipitation (ChIP): Chromatin of HEK293 cells was cross-linked with 1% formaldehyde and the reaction was terminated by adding 0.125 M glycine for 5–10 min. Cell pellets were lysed in SDS lysis buffer on ice for 20 min (1% SDS, 10 mM EDTA, 50 mM Tris-HCl pH 8.1, 300 µL/2–3 × 106 cells) and sonicated using a Bioruptor^®^ Pico sonicator (Diagenode, Denville, NJ, USA), with 10 cycles at high intensity, 30 s on and 20 s off. Sheared chromatin samples were diluted 1:10 (0.01% SDS, 1.1% Triton, 1.2 mM EDTA, 16.7 mM Tris-HCl pH 8.1 and 167 mM NaCl), protein A/G Magnetic Beads (Thermo Scientific) were added and incubated at 4 °C for 20 min. Pellets were washed twice with 1 mL of each low salt buffer (0.1% SDS, 1% Triton, 2 mM EDTA, 20 mM Tris-HCl pH 8.1, 150 mM NaCl), high salt buffer (0.1% SDS, 1% Triton, 2 mM EDTA, 20 mM Tris-HCl pH 8.1, 500 mM NaCl), LiCl buffer (0.25 M LiCl, 1% NP40, 1% deoxycholate, 1 mM EDTA, 10 mM Tris-HCl, pH 8.1) and TE buffer (pH 8.1), followed by centrifugation (5000× *g* for 1 min at 4 °C). Chromatin was eluted with 250 µL of nuclease-free water. Reverse cross-linking was performed with proteinase K followed by phenol/chloroform extraction and ethanol precipitation. qPCR was carried out using Sybr Green I Master mix (Roche) in a LightCycler^®^ 480 System (Roche) with the following parameters: pre-incubation at 95 °C for 10 min; initial denaturation at 95 °C for 1 min; denaturation at 95 °C for 10 s; annealing at 57 °C for 30 s; and extension at 72 °C for 30 s for 45 cycles. All ChIP-qPCR primers are listed in Table S3. Data were normalized to rabbit anti-IgG precipitated material. The fold change (R) was determined using the formula:

R = 2^(Ct of H3K27Ac/H3K4Me3 − Ct of IgG)^

CRISPR-Cas9: *SLC34A1*-sense/antisense directed sgRNAs were designed using web tools (https://portals.broadinstitute.org/gpp/public/analysis-tools/sgrna-design, accessed on 24 July 2017) (Table S4). In essence, the protocol by the Zhang lab was followed [58]. Oligonucleotides were annealed, phosphorylated using T4 polynucleotide kinase (New England Biolabs), ligated into *Bbs*I linearized pSpCas9 (PX458) (Addgene, Watertown, MA, USA), followed by transformation of Stbl3 E. coli (Thermo Fisher). Recombinant plasmids were isolated using Plasmid Mini Kit II (Bioline, Toronto, ON, Canada), and inserts were confirmed by DNA Sanger sequencing using primer pSp-R (GATC Biotech). Transfection efficiency using HEK293 cells and Lipofectamine 2000 (Invitrogen) was tested, followed by the detection of nicked DNA to evaluate the efficiency of the different guide RNAs (EnGen Mutation Detection kit, New England Biolabs). Primers were designed to amplify a genomic region >1000 bp (Table S4). PCR products were treated with T7-endonuclease I (New England Biolabs) and separated on a 2% agarose gel, band intensity was quantified using a ChemiDoc XRS+ Imaging System (BioRad, Hercules, CA, USA).

The homology repair template was designed with gene-specific arms flanking the CRISPR-Cas9 cleavage site, a transcriptional termination cassette with a puromycin antibiotic selection marker driven by the CMV promoter and a transcriptional terminator sequence, BGH-PolyA (bovine growth hormone polyadenylation) (Figure 4B). All components were amplified by PCR (primers listed in Table S5) and assembled using the NEBuilder^®^ HiFi DNA Assembly Cloning Kit (New England Biolabs) at 50 °C overnight. Assembled fragments were examined on a 1% agarose gel, re-amplified and purified using the GeneJET PCR Purification Kit (Thermo Scientific, Waltham, MA, USA). In total, 500 ng of DNA (sgRNA containing plasmid and HDR repair template) was used to co-transfect HEK293 cells followed by cloning by serial dilution in the presence of puromycin (1 µg/mL). The genomic DNA of individual clones was purified using a Wizard^®^ Genomic DNA Purification Kit (Promega) followed by genotyping for integration of the HDR cassette within *SLC34A1*-sense and -antisense genomic regions. PCR was performed using GoTaq^®^ Long PCR 2 × Master Mix with the following conditions: denaturation at 94 °C for 30 s; annealing 59 °C for 30 s; extension at 65 °C for 4 min (primer sequences, Table S6). PCR products were separated on a 1% agarose gel and clones which showed mono- or biallelic HDR cassette insertion were confirmed by sequencing (GATC Biotech).

^32^P uptake measurements: HEK293 cells were treated with dexamethasone (100 nM) for 5 days prior to uptake measurements, with replacement of the drug containing growth medium every 48 h. On the day of the 32P uptake experiment, the number of cells was counted from one well for each treatment condition using a Cellometer Auto T4 Bright Field Cell Counter (Nexcelom, Lawrence, MA, USA). For uptake, the cells were washed gently three times with 500 µL of warm phosphate-free Kreb’s buffer (140 mM NaCl, 5.4 mM KCl, 1.2 mM, MgSO_4_, 4 mM glucose, 2 mM CaCl_2_, 10 mM HEPES/Tris pH 7.4) and adapted to Kreb’s buffer on a thermostat-controlled platform at 37 °C for 30 min. Cells were then exposed to 1 mM phosphate containing 1 µC/mL ^32^P tracer for 5 min, followed by three washes with ice cold Kreb’s buffer. Cells were lysed with 250 µL of 0.1% SDS (*v/w*) and ^32^P was quantified by scintillation counting (Beckman LS5000).

Xenopus oocyte expression: Oocytes were purchased from the European Xenopus Resource Centre (Portsmouth, UK) and defolliculated with collagenase using standard procedures [59]. Oocytes were injected with 50 ng of total RNA isolated from HEK293 cells as described above. Cells were incubated for 3–5 days in modified Barth’s solution. Uptake was performed as described using ^32^P as a tracer [60].

Statistical analysis: Unpaired student *t*-testing was used to compare pyrosequencing data. For RT-qPCR data, one-way ANOVA followed by Games–Howell Post-Hoc test or Tukey’s test for multiple comparisons was performed using GraphPad Prism version 6.00 (GraphPad Software, San Diego, CA, USA). Values of *p* < 0.05 were considered as significant, unless otherwise mentioned. Data are presented as mean ± standard error of the mean (SEM).

## 5. Conclusions

We found that the bi-directionally transcribed *SLC34A1/PFN3* locus can be activated using epigenetic drugs such as zebularine and dexamethasone in human cell lines. The effect of the drugs depended on the cellular background. In HEK293 cells, both sense (*SLC34A1*) and antisense transcripts (*PFN3*) were maximally induced after 5 days’ incubation with dexamethasone. The transcriptional induction was paralleled by changes in DNA methylation and H3K27 acetylation in the sense promoter. Co-expression of transcripts from the opposite strand could also be demonstrated by CRISPR-mediated activation of the sense and the antisense transcript, respectively. These findings indicate that the *SLC34A1* sense transcript and the *PFN3* antisense transcript are regulated in a concordant way. Based on these findings, we propose a model whereby transcription in sense direction induces low-level production of the non-protein coding *PFN3* RNA. Such ‘co-expression’ produces comparably high levels of protein coding sense transcript and low levels of the corresponding antisense transcript. The antisense transcript may adopt regulatory roles and function as lncRNAs in a cell-specific manner. Since low-level expression and concordant regulation with the cognate sense mRNA are hallmarks of many complementary gene pairs [8], our findings may apply to other sense/antisense genes.

## Data Availability

Not applicable.

## Supplementary Materials

The following supporting information can be downloaded at: https://www.mdpi.com/article/10.3390/ncrna8010019/s1, Figure S1: DNA methylation analysis of *SLC34A1*-sense (S1, S2) and *SLC34A1*-antisense (AS) promoters; Figure S2: Global histone H3 acetylation; Figure S3: PCR analysis of CRISPR edited HEK293 clones; Tables S1–S6: PCR primers.
