# Supplementary material for: Interdependent Transcription of a Natural Sense/Antisense Transcripts Pair (SLC34A1/PFN3)
Source: Noncoding RNA. 2022 Feb 11;8(1):19. doi: 10.3390/ncrna8010019 (PMC8877773; doi:10.3390/ncrna8010019)
Supplement: Supplementary file 1 [file ncrna-08-00019-s001.zip › ncrna-1532055-supplementary/ncrna-1532055-supplementary.pdf]

A) DNA methylation in HKC-8 cells

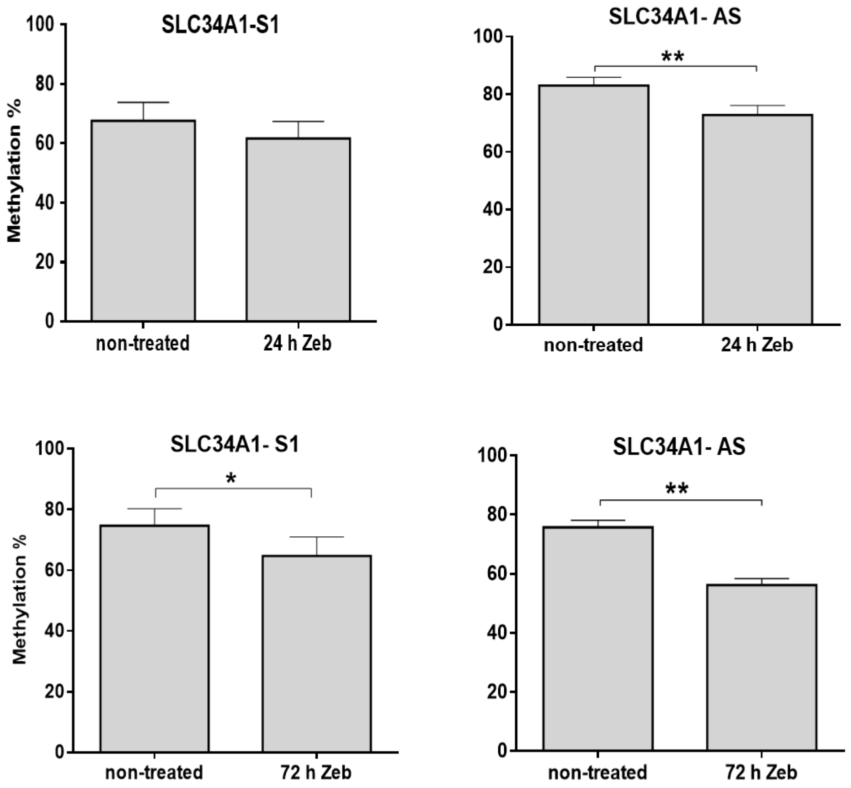

B) DNA methylation in HEK293 cells

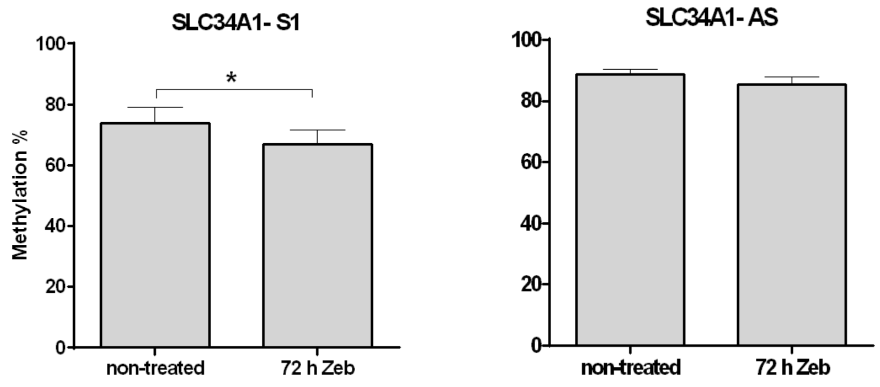

Supplementary Figure S1: DNA methylation analysis of SLC34A1-sense (S1, S2) and SLC34A1-antisense (AS) promoters. A) HKC8 cells treated for 24 and 72 h with 50 µM zebularine. B) HEK293 cells treated for 72 h with 50 µM zebularine. Statistical analysis for comparing the treated cells with the control was performed by unpaired students t-test, (*= p<0.05 and **= p<0.01).

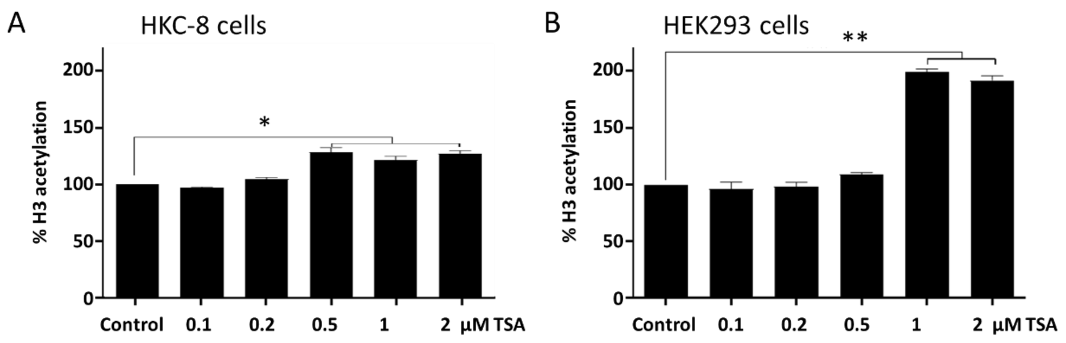

Supplementary Figure S2: Global histone H3 acetylation. Histone H3 were purified and acetylation was quantified using a total histone H3 acetylation detection kit (EpiGentik). (**A**) HKC-8 and (**B**) HEK293 cells show a dose-dependent response to TSA. The data were normalised to control cells (non-treated) and treatment conditions were compared by One-Way ANOVA test using Tukey's test (* $p < 0.05$, ** $p < 0.01$).

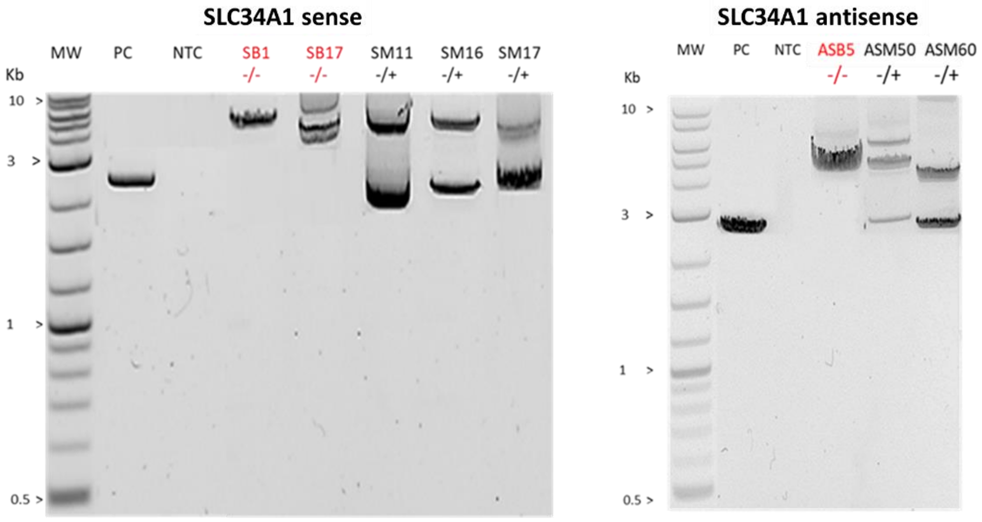

Supplementary Figure S3: PCR analysis of CRISPR edited HEK293 clones. Clones were isolated by serial dilution in puromycin containing selection medium and genomic DNA was isolated followed by end-point PCR. For the SLC34A1 sense transcript, the expected amplicon is 2222 bp or 4112 bp with the integrated cassette. Similarly, the SLC43A1-antisense derived amplicon is 2425 bp and 4315 bp with HDR cassette. Agarose gel of PCR products including a positive control, PC (unedited HEK293) and a negative control, NTC (PCR without addition of no DNA). Sense bi-allelic (SB) clones SB1 and SB17 show bi-allelic integration (-/-, red), three clonal cells show mono-allelic integration (-/+). C) SLC34A1-antisense clone ASB5 with bi-allelic insertion (-/-, red), ASM50 and ASM60 with mono-allelic insertion (-/+).

Supplementary Table S1: Primer sequences and product length for RT-qPCR of SLC34A1 sense/antisense and GAPDH.

Oligo ID	Sequence (5'-3')	Amplicon (bp)
**SLC34A1-F**	CAGCCCTCAGGTCCTACACA	163
**SLC34A1-R**	CTCTGGCTTCTGCTCCTCCT	
**SLC34A1-AS-F**	TGGGCGACTGGAAGGTCTACA	182
**SLC34A1-AS-R**	GTCTTCGGCACCACCACAATTA	
**GAPDH-F**	TGAAGGTCGGAGTCAACGGATTTG	128
**GAPDH-R**	CATGTAAACCATGTAGTTGAGGTC	

Supplementary Table S2: Pyrosequencing primers. All primers were designed using MethPrimer webtools and synthesised by IDT, UK.

Oligo ID	Orientation	Sequence (5'-3')	bp	No. of CpG
**AS1**	F	TTTGTAGGAAGGTGTGTTTGTT	173	3
**AS1-(bio)**	R	CTAAAAAATCTACATCAATACAATAC		
**AS1**	SP	TTTGTAGGAAGGTGTGTTTGTT		
**AS1**	Sequencing entry	C/TGGTTTC/TGTGAGTAC/TGTTTATTTTTTGC/TGGC/TGAGATGGTC/TGTTAGTAGGTT		
**AS2**	F	GTATTGTATTGATGTAGATTTTTTAG	461	5
**AS2- biotin**	R	CCTAAACAACATAATAAAACCTCATCTC		
**AS2**	SP	TTGTGAGGTAGGTTGGG		
**AS2**	Sequencing entry	C/TGGGGTTGTGAC/TGTTATATAAGGC/TGTC/TG		
**SLC34A1-S1-**	F	AGTGGGAGTAAATTTTTATGGAGGT	339	7
**SLC34A1-S1biotin**	R	CATCCCAACAAACTAAAATCAAAA		
**SLC34A1-S1-**	SP	AGTGGGAGTAAATTTTTATGGAGGT		
**SLC34A1-S1-**	Sequencing entry	C/TGTTTTTTTTAGTGTTTTC/TGC/TGGAGATTC/TGTTTTTTTAGTGTTTTC/TGC/TGGAGGTTC/TGTTTTTTTAG		
**SLC34A1-S2-**	F	TATAGGATGTTTGGGTTATTTTTTG	148	2
**SLC34A1-S2biotin**	R	CTATAATCAAATTCTTCCCATCAAC		
**SLC34A1-S2-**	SP	GTTTTTGTTTGTAGG		
**SLC34A1-S2-**	Sequencing entry	C/TGGGGGATGTGTTTGGGTC/TGTGGTTG		

Supplementary Table S3: Primers for ChIP-qPCR and product length. Amplicons cover the SLC34A1-sense and -antisense promoters and an SLC34A1-sense internal enhancer (i.e. SLC34A1-B).

Oligo ID	Sequence (5'-3')	Amplicon (bp)
**SLC34A1-A-F3**	GCCATAGGATGTTTGGGTCA	167
**SLC34A1-A-R3**	AGCTCTGCATGGGGAATCTA	
**SLC34A1-B-F2**	GTGTTTGTGCGTGTGAGTCT	193
**SLC34A1-B-R2**	CCACTATGAGGTCTGCAGGT	
**AS-SLC34A1-F3**	TATAGGATCGTGTCGCTGCA	193
**AS-SLC34A1-R3**	ACGCCTGTAATCTCGTCACT	

Supplementary Table S4: Oligonucleotide sequences used for the T7-endonuclease test.

Oligo ID	Sequence (5'-3')	Oligo ID	Sequence (5'-3')
**pSp-R**	TAGGGGGCGTACTTG GCATATG		
**S-g-1F**	GTGAGGGTGTGTGGG CATGAGT	**AS-T7E1234 F**	CTCTAAATTCACCCAC CTCAG
**S-g-1R**	GAGCAGACGAAG AGGTAGAG	**AS-T7E1234 F+**	CAGCCACACATCACTC CTTCCTG
**S-g-2F**	CTCTACCTCTTCGTC TGCTC	**AS-TE71234 R**	CATCAGGGTGTCAGG CTGGAG
**S-g-2R**	CAG CCT TACTGG GGTCACGCT	**AS-TE71234 R+**	CTAGGGACTCCGCAG CTGAG
**S-T7E1R**	CAGGGGCAGGTGTG CTCAGC	**AS-T7E5678 F**	CTCAAAATCCCGGCTC CACCAG
**S-T7E123 F+**	CAAGCCTCTGGTTCG GAGC	**AS-T7E5678 F+**	CAAGTGGAGGAGGAG CCAGC
**S-T7E123 F++**	CTGGGCGTCCTCAGT TCTC	**AS-T7E5678 R**	CATGCGGACAACAGCT GCGTG
**S-T7E123 R+**	GTCCAGGGAGCAGAC GAAGAG	**AS-T7E5678 R+**	GAAGGTCTACATCAGT GCAGTG
**S-T7E123 F**	CTGGGCTAGAGCTCA ATAATAC	**AS-T7E1234 F**	CTCTAAATTCACCCAC CTCAG
**S-T7E23 R**	GTGTAGGACTAGGGA GGGAG	**AS-T7E1234 F+**	CAGCCACACATCACTC CTTCCTG

Supplementary Table S5: Primers used to generate the HDR template. Lowercase letters show the overlapping region with adjacent fragments. S and AS refers to SLC34A1-sense and-antisense transcripts, respectively.

Oligo ID	Sequence (5'-3')
S-PCR1-F	CACCGCGCCCCATATAACATGTC
S-PCR1-R	CAATGCCCTGGCCTACGGATTC
S-PCR2-F	CAACTCTTCACACCCCTACAAC
S-PCR2-R	caaatgtaacgcgtctcatagGACAACATCCTGGGCACCCAC
S-PCR3-F	gttacattttgctcacgtctcgcTACTACGGAGAGAGGCTGGGG TCCCCTGCTG
S-PCR3-R	cattttgctcacgtctcgctacTACGGAGAGAGGCTGGGGTC
S-PCR4-F	gtgggtgcccaggatgttgTCCTATGAGACGCGTTACATTTG
S-PCR4-R	gaccccagcctctctccgtagTAGCGAGACGTGAGCAAAATG
AS-PCR1-F	GAGTTCCTCCCTCTAGTCCAG
AS-PCR1-R	CTGGGCAACATAGTGAGACCTC
AS-PCR2-F	CAGCACCATCTCCCCTCAAAGAC
AS-PCR2-R	gtaacgcgtctcataggggggGCATCCTCAACAAGAC
AS-PCR3-F	tgttgaggatgccccccTATGAGACGCGTTACATTTG
AS-PCR3-R	gacgcggcgtacattagCGAGACGTGAGCAAAATG
AS-PCR4-F	ctcacgtctcgctaATGTACGCCGCGTCGG
AS-PCR4-R	CTCGCTCTGTGGCTCAGGCT

Supplementary Table S6: SLC34A1-sense/antisense genotyping PCR primers and amplicon length.

Oligo ID	Sequence (5'-3')	Amplicon (Knock-in)	Amplicon (control)
**Sense-F**	CACCGCGCCCCATATAACATGTC	4112	2222
**Sense-R**	CAATGCCCTGGCCTACGGATTC		
**Antisense-F**	CATGGGACTTCCTGCCTCGCTG	4315	2425
**Antisense-R**	CATCGTGGGCCATGCGGACA		
